# Supplementary material for: Epigenetic features drastically impact CRISPR–Cas9 efficacy in plants
Source: Plant Physiol. 2022 Jun 11;190(2):1153–64. doi: 10.1093/plphys/kiac285 (PMC9516779; doi:10.1093/plphys/kiac285)
Supplement: kiac285_Supplementary_Data [file kiac285_supplementary_data.zip › 00291suppdata.pdf]

Supplemental Information

Epigenetic features drastically impact CRISPR-Cas9 efficacy in plants

Trevor Weiss[1,3,5,6], Peter A. Crisp[1,2,3], Krishan M. Rai[1], Meredith Song[4], Nathan M. Springer[1,3], Feng Zhang[1,3,5,6]*.

[1]Department of Plant and Microbial Biology, University of Minnesota, Saint Paul, MN 55108
[2]School of Agriculture and Food Sciences, The University of Queensland, Brisbane QLD 4072, Australia
[3]Center for Precision Plant Genomics, University of Minnesota, Saint Paul, MN 55108
[4]Department of Genetics, Cell Biology and Development, University of Minnesota, Minneapolis, MN 55108
[5]Microbial and Plant Genomics Institute, University of Minnesota, Minneapolis, MN, 55108
[6]Center for Genome Engineering, University of Minnesota, Minneapolis, MN, 55108

Corresponding author: Feng Zhang

Email: zhangumn@umn.edu

This file includes:

Figures S1 to S9
Tables S1 to S3
Legends for Datasets S1 to S4
SI References

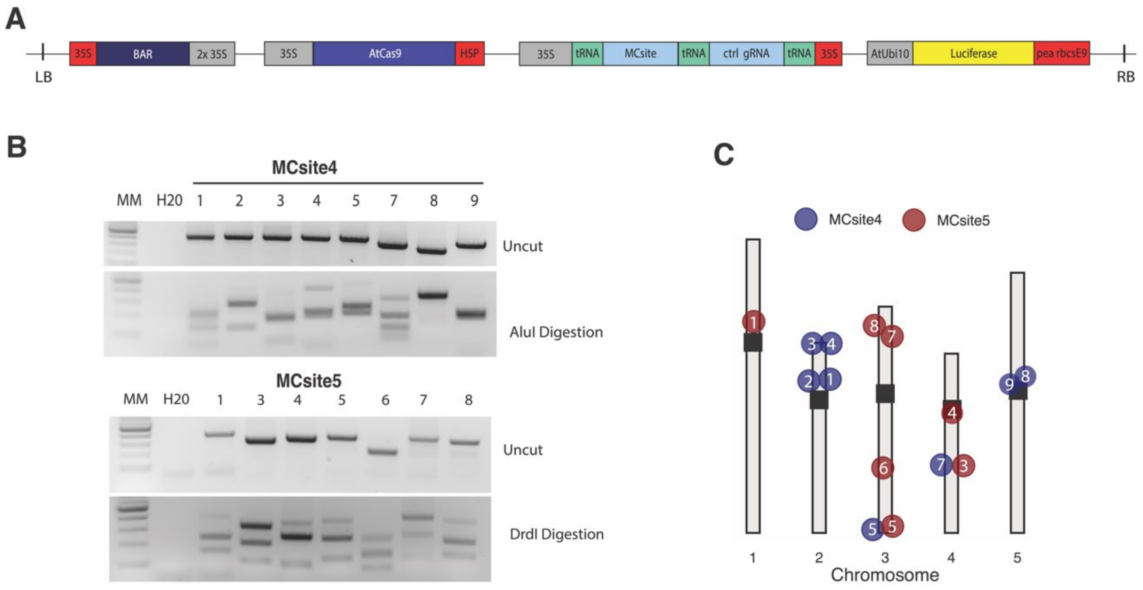

Supplemental Figure S1. Characterization of multicopy CRISPR sites (MCsites) for CRISPR-Cas9 mutagenesis. (A) Illustration of the transfer DNA (T-DNA) constructs with left border (LB) and right border (RB) on each end . (B) Representative cleaved amplified polymorphic sequence (CAPS) genotyping images for MCsite4 and MCsite5. Samples were genotyped by genomic PCR (Uncut) and the CAPS assay (Alu Digestion for MCsite4 and DrdI digestion for MCsite5), with a 1-kb ladder (MM), and no genomic DNA control (H20). (C) Distribution of the CRISPR target sites of MCsite4 (blue) and MCsite5 (red). Gray bars represent each chromosome with the black box indicating the centromere. The white number inside of each colored circle corresponds to the CRISPR target for that MCsite. Site 6 from MCSite4 and Site 2 from MCSite5 were not amplifiable with the site-specific PCR primers. Thus, they were excluded from the CAPS and NGS assays.

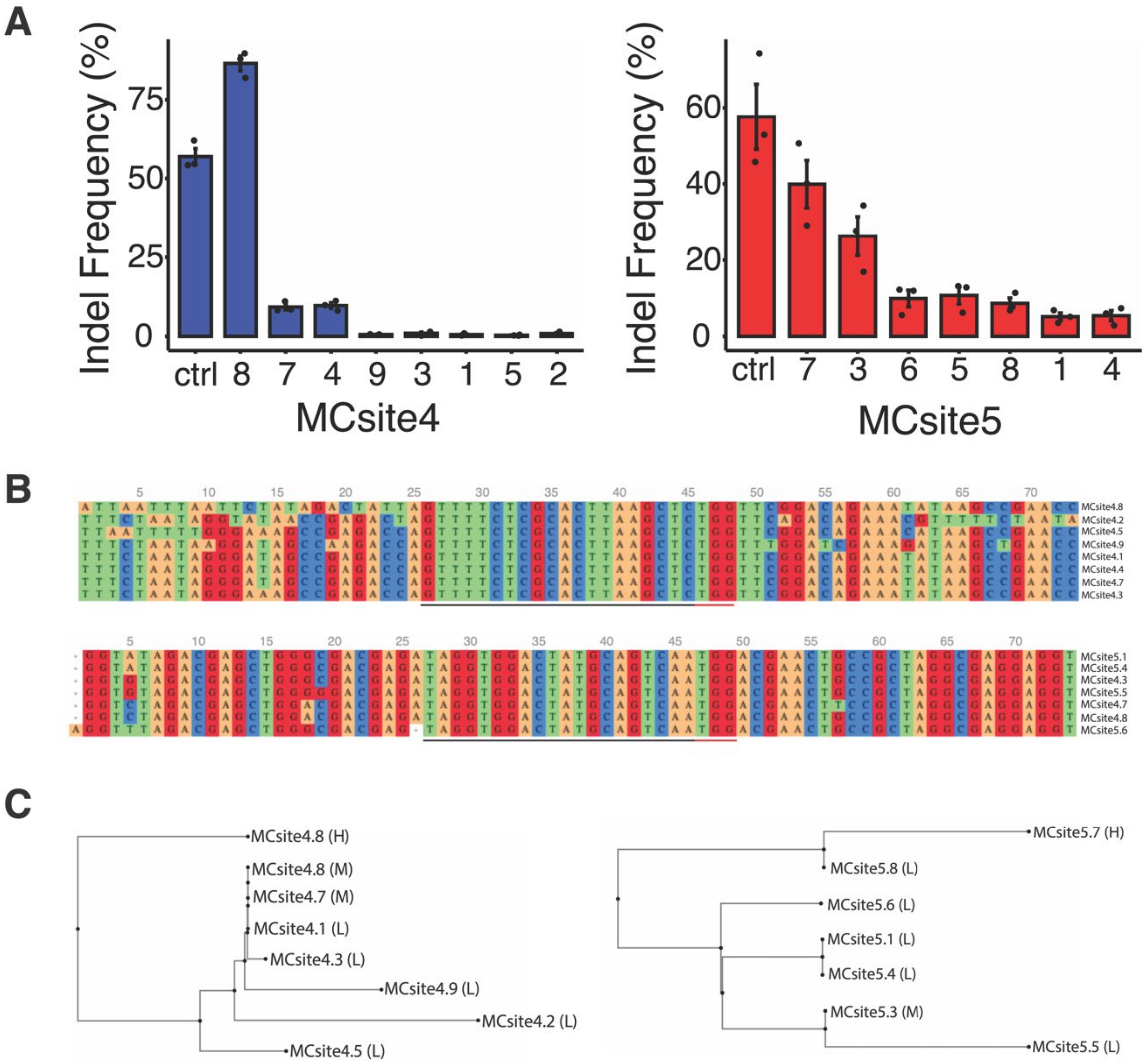

Supplemental Figure S2. Non-normalized mutagenesis efficiency and sequence comparison for individual target sites in multicopy CRISPR site 4 (MCsite4) and multicopy CRISPR site 5 (MCsite5). (A) Bar graphs displaying the non-normalized mutagenesis frequencies at CRISPR targeted sites for MCsite4 (blue) and MCsite5 (red). Ctrl is abbreviated for the CHLI2 site. The non-normalized indel frequency was calculated by dividing the number of edited reads by the number of total reads, for each replicate. The standard error (SEM) is displayed for each target site with the dots indicating independent replicates (n = 3). (B) Sequence alignments of the CRISPR targets for MCsite4 and MCsite5. The sequence includes the 25 nucleotides to the left and to the right of the protospacer (underlined by a black bar) and PAM sequence (underlined by a red bar). The dendrogram indicates similarity between the sequences. Alignment was created using the MAFFT version 7 online tool with default settings (https://mafft.cbrc.jp/alignment/server/) (1). (C) Sequence similarity dendrograms were created using the Neighbor-Joining (NJ) method on MAFFT version 7 online tool with default settings (https://mafft.cbrc.jp/alignment/server/). H (high), M (moderate), and L (low) next to each target site indicate the mutagenesis group that CRISPR site is associated with.

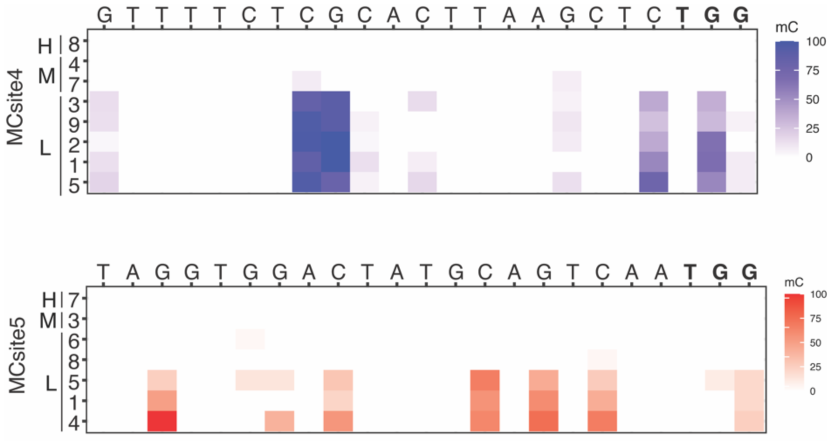

Supplemental Figure S3. Single nucleotide heatmap of DNA methylation levels at multicopy CRISPR site (MCsite) 4 (blue) and 5 (red) protospacer and PAM (bold) sequences from 0 (unmethylated) to 100 (fully methylated). H (high), M (moderate), and L (low) along the y-axis indicate which mutagenesis group that CRISPR site is associated with. The PAM sequence is bold.

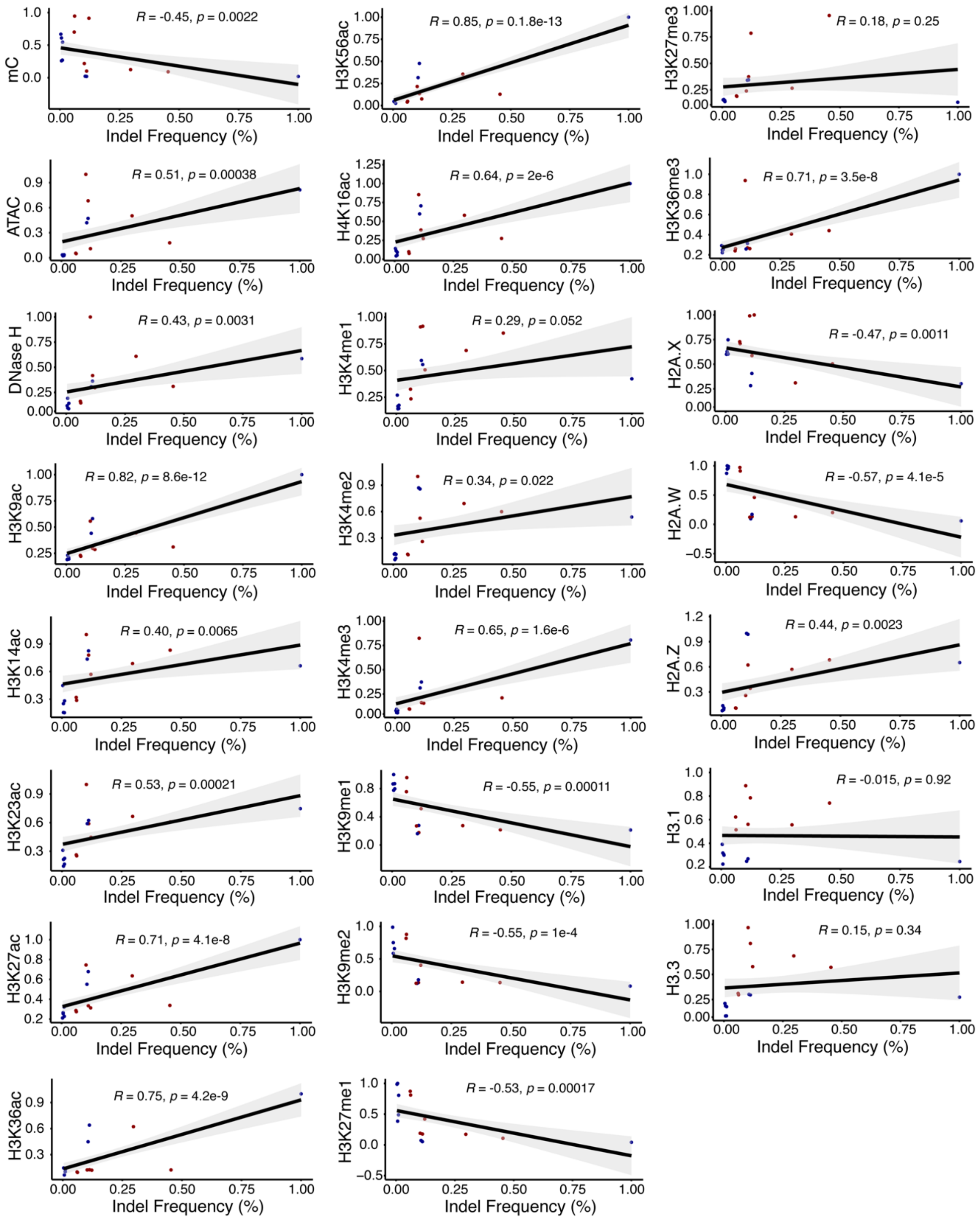

Supplemental Figure S4. Correlation analysis for CRISPR-Cas9 mutagenesis frequencies and chromatin features. The blue dots represent multicopy CRISPR site 4 (MCsite4) and the red dots represent MCsite5. The trendline is black with gray indicating the standard error. The R value and p-values are indicated at the top of each correlation plot according to Spearman's rank correlation coefficient. Each feature was normalized using all 15 target sites on a scale of 0 to 1, with higher values indicating the higher levels for the respective feature.

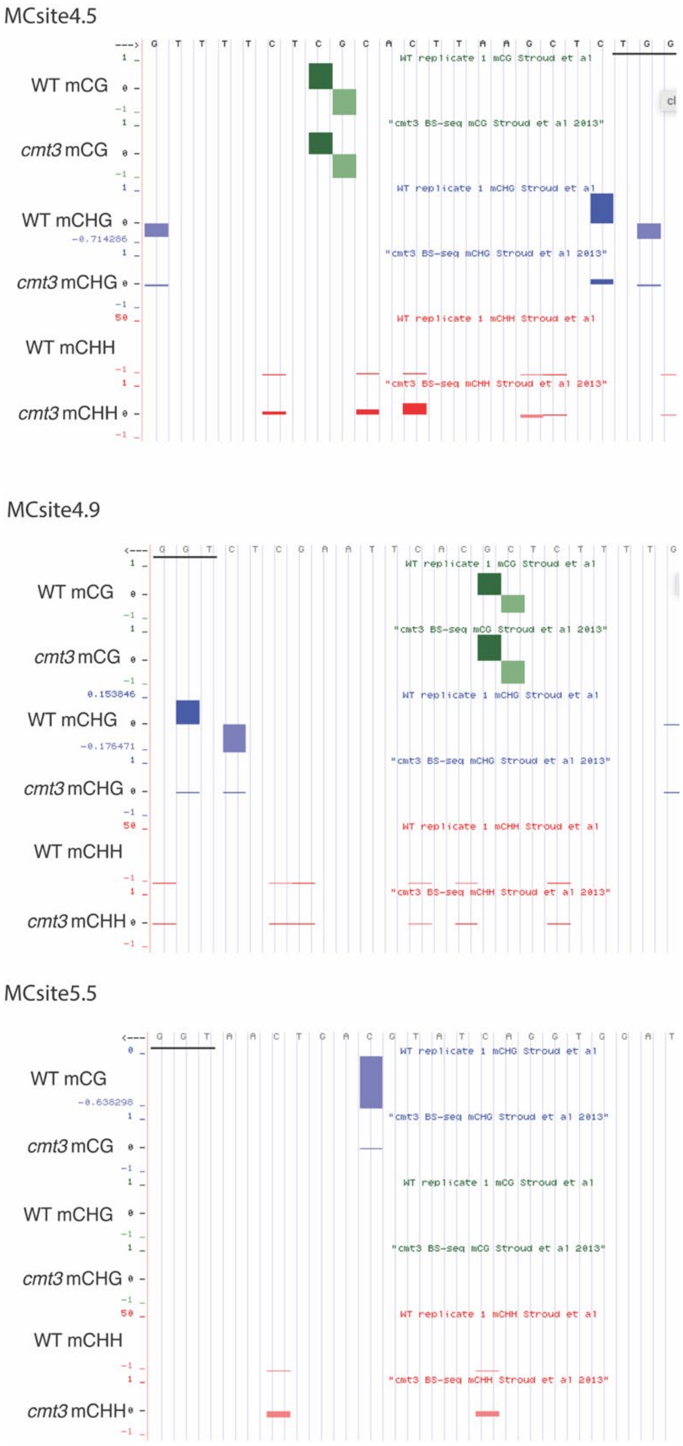

Supplemental Figure S5. Characterization of the single-based DNA methylation status at multicopy CRISPR site (MCsite) 4 and 5 in the wild type and *cmt3* mutant plants. Representative screenshots of MCsite4.5, MCsite4.9, and MCsite5.5 from the UCSC genome browser (2). The protospacer sequence is displayed along the top of each screenshot with the PAM underlined. 6 individual methylome tracks (wild type mCG context, cmt3 mCG context, WT mCHG context, cmt3 mCHG context, WT mCHH context, and cmt3 mCHH context) displaying the levels of DNA methylation at each individual nucleotide along the protospacer and PAM genomic DNA sequence. Green corresponds with mCG, blue with mCHG and red with mCHH.

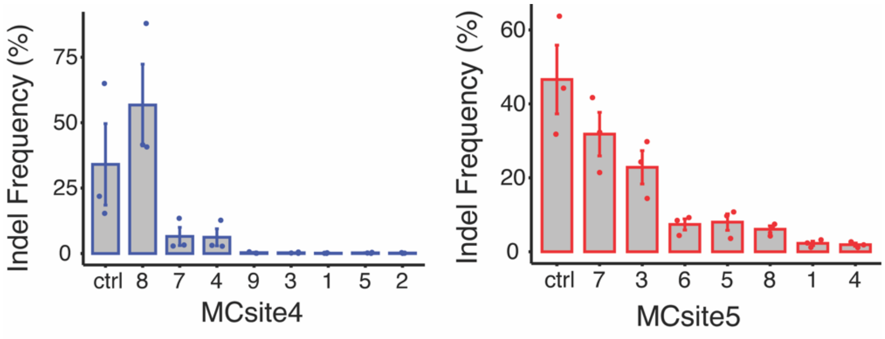

Supplemental Figure S6. Unnormalized mutagenesis frequencies for multicopy CRISPR site (MCsite) 4 (blue and gray) and 5 (red and gray) in the *cmt3* mutant plants. Ctrl is abbreviated for the CHLI2 site. The standard error (SEM) is displayed for each target site with the dots indicating independent replicates (n = 3).

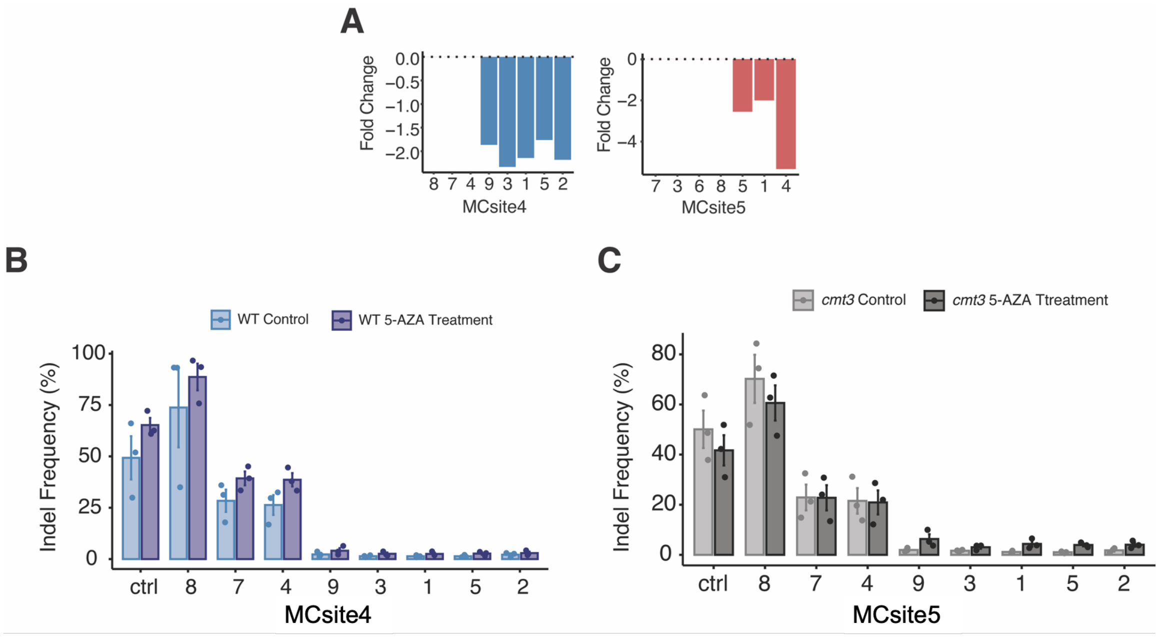

Supplemental Figure S7. 5-Azacytidine treatment of the wild type and *cmt3* T2 seedlings. (A) DNA methylation reduction fold changes in a 1kb window (500 bp upstream and downstream from the CRISPR-Cas9 cut site) in the 100μM treated 5-azacytidine samples relative to the mock untreated samples from (3). Fold change was calculated by dividing the WT mock nontreated value by the 5-AZA 100 μM value, and then multiplied by -1. The dotted line at the top of each bar graph represents zero change, and each bar is color coded as either blue (MCsite4) or red (MCsite5). (B) and (C) Unnormalized mutagenesis frequencies for MCsite4 and the CHIL2 control (ctrl) in the wild type plants with and without 5-azacytidine treatment (B), and in the cmt3 mutant plants with and without 5-azacytidine treatment (C). The standard error (SEM) is displayed for each target site with the dots indicating replicates (n = 3).

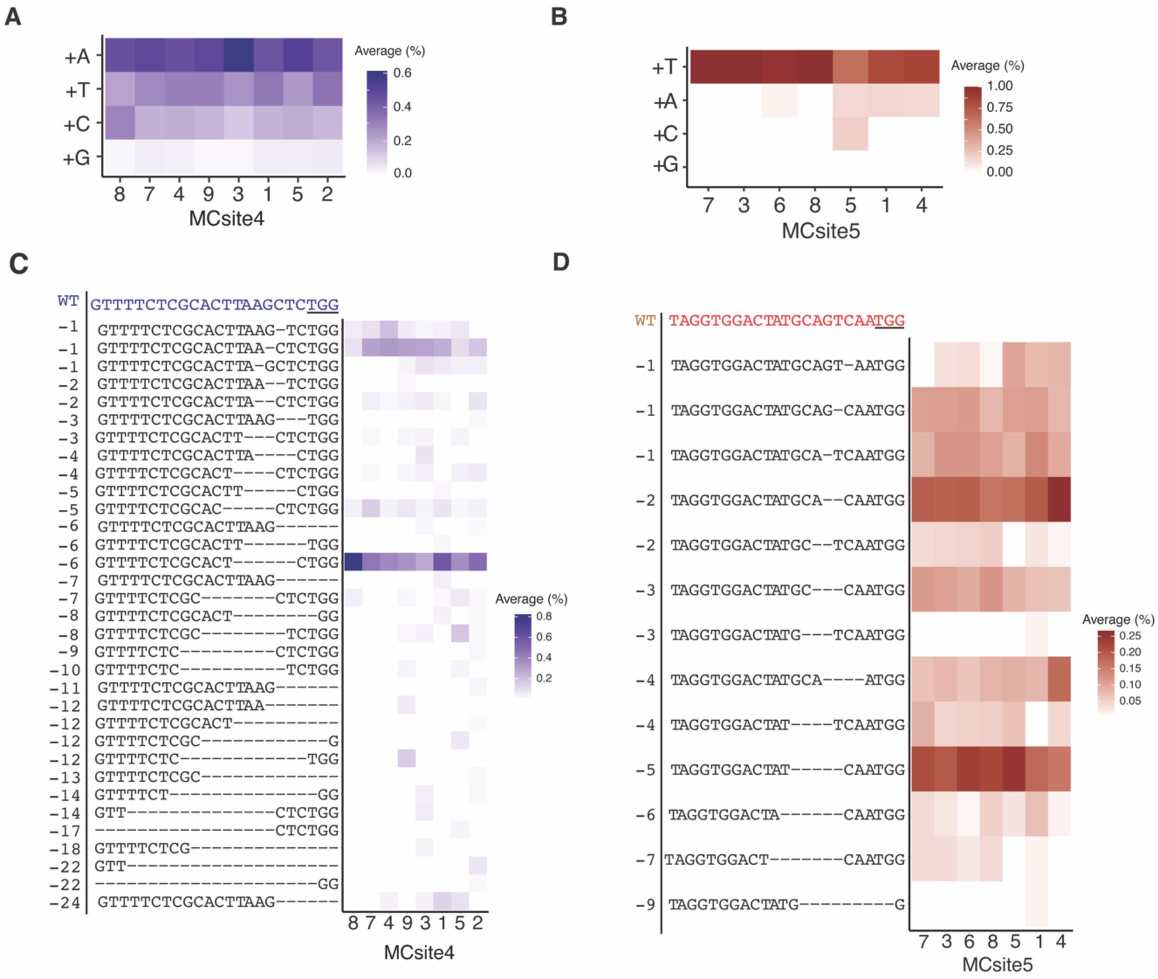

Supplemental Figure S8. Characterization of mutation outcomes for multicopy CRISPR site (MCsite) 4 (blue) and 5 (red). (A) and (B) Heatmap displaying the frequency of 1 bp insertions that occurred at each MCsite4 (A) and MCsite5 (B) sites in wild type plants. In the MCsite4 sites, the majority of 1 bp insertions (A, T or C) was derived from template-independent DNA polymerase-mediated end filling. In the MCsite5 sites, the majority of 1 bp insertions (T) was derived from templated-dependent end filling. (C) and (D) Heatmap displaying the frequency of deletion outcome that occurred at each MCsite4 (C) and MCSite5 (D) sites in wild type plants. The wild type sequence is at the top of the y-axis with the PAM underlined. The number to the left of each deletion outcome indicates the size of the deletion. The frequency of each repair outcome was calculated by using the total number of reads with insertions or deletions divided by the total number of mutated reads for each site. This was done for all three replicates. The average of the three replicates was then calculated and plotted as a heatmap.

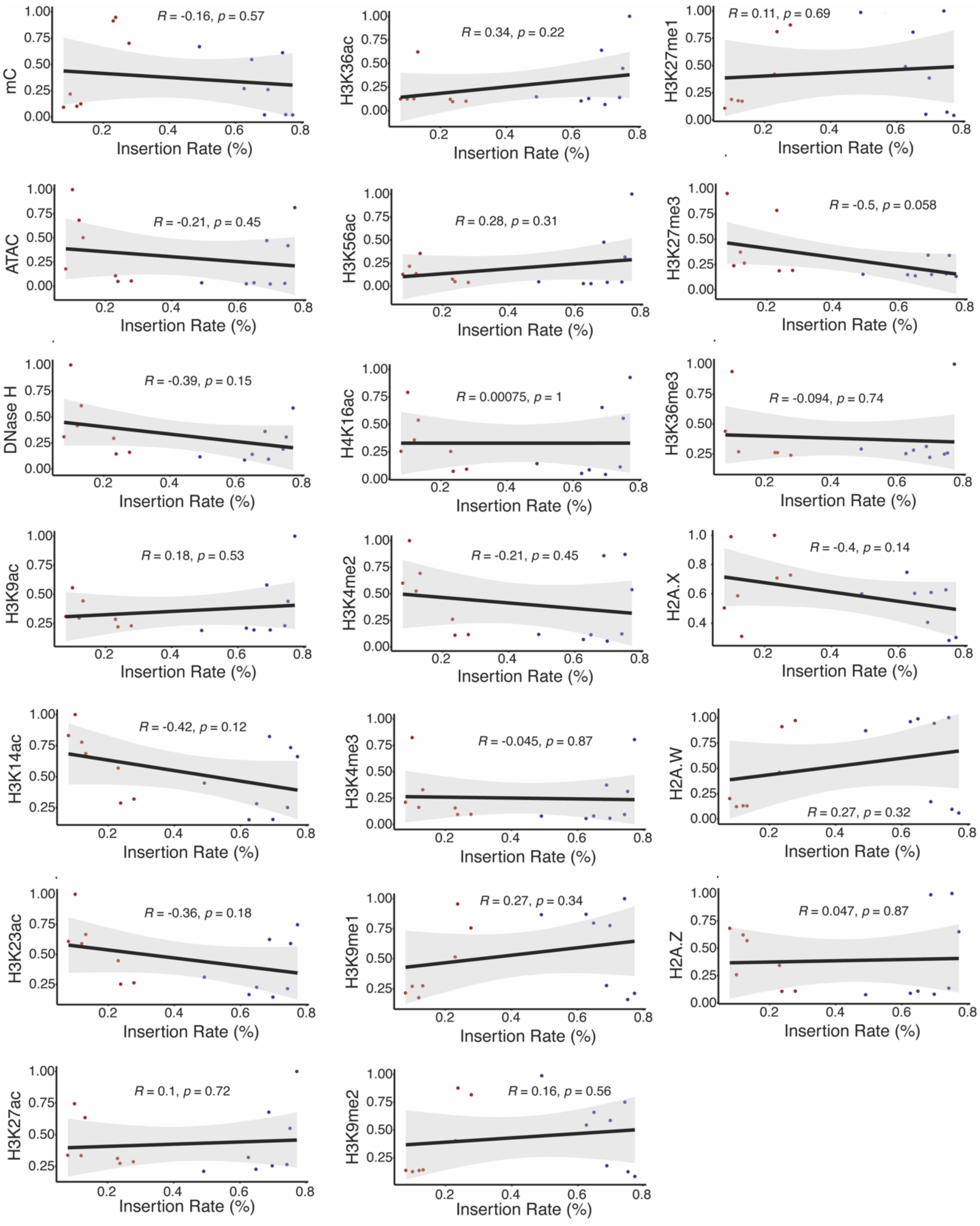

Supplemental Figure S9. Correlation analysis for 1 bp insertion rate and chromatin features. The blue dots represent MCsite4, and the red dots represent MCsite5. The trendline is black with gray indicating the standard error. The R value and *p*-values are indicated at the top of each correlation plot according to Spearman's rank correlation coefficient. Each chromatin feature was normalized using all 15 target sites to allow for comparison between the CRISPR target sites on a scale of 0 to 1, with higher values indicating the higher levels for the respective chromatin feature.

Supplemental Table S1. Primer sequences to amplify each CRISPR target site analyzed in these experiments.

target site	primer name	primer sequence
CHLI2	119_F2_CHLI2	GTCCCATCTCTGCTTCGGAC
	120_R1_CHLI2	CACACCACCTATCTTCGGGT
MCsite4.1	330_F_1_gRNA_5907	ACGAATGGCTTGATCCCTTC
	334_R_1_gRNA_5907	TTCTGGGCTCTGTTTCAAGAATA
MCsite4.2	339_F_2_gRNA_5907	TCCTTGGAATGCTGTTCACC
	343_R_2_gRNA_5907	CCCTTGTAATGGCTACCGATC
MCsite4.3	348_F_3_gRNA_5907	AATACGAACTACGAATGGCTTGA
	351_R_3_gRNA_5907	TCCAGCAGGCTTATGTATGATTT
MCsite4.4	356_F_4_gRNA_5907	ACTGGGTACGTAGGCAATCC
	362_R_4_gRNA_5907	TAAAAGTATTCGGCGTGTACTGG
MCsite4.5	143_F_5_5907	GCAGATCGCCTATCGCAAGG
	374_R_5_gRNA_5907	TGGACTAGATGTTAGGTGCGA
MCsite4.7	146_F_7_5907	AATCCGACCTCTCCGTTAGAACT
	142_R_7_5907	AAAATAAGTGAGGCGGACTCAGCA
MCsite4.8	394_F_8_gRNA_5907	CGGTTTATGTTGATCGGATTTTA
	397_R_8_gRNA_5907	AAAGAGGGAGTTCTGGGCTC
MCsite4.9	403_F_9_gRNA_5907	GTCATTCTCAAGGATGCAGCTAT
	407_R_9_gRNA_5907	AGACTTAGCTCGGCTTTTTCAAT
MCsite5.1	195_F_1_gRNA 6169	AAGTGAGCTAGGCCGTCATT
	196_R_1_gRNA 6169	ACACCCAGAGACAGAGTAGC
MCsite5.3	201_F_3_gRNA 6169	GGCTGCCTACGTACCCTCAGAAA
	202_R_3_gRNA 6169	GCTCGTCTTCAAGCTCTTCCTCAG
MCsite5.4	197_F_4_gRNA 6169	GGTAGGTAGATGTGCGACGA
	198_R_4_gRNA 6169	GGACACCCAGAGAGAGTAGC
MCsite5.5	199_F_5_gRNA 6169	TAGGTCGGCAGGTAGTTGAC
	200_R_5_gRNA 6169	CTCGTTAGCTCGCTCCTCAT
MCsite5.6	203_F_6_gRNA 6169	CTGGATGAACAGTGGTAGAGAGGCG
	204_R_6_gRNA 6169	TCTTCAAGCTCTTCCCATCACTCGA
MCsite5.7	205_F_7_gRNA 6169	AGGTAGGTCGGCAGGTAGGT
	206_R_7_gRNA 6169	TTAGCTCGCTCCTCTTCGTCAAGC
MCsite5.8	207_F_8_gRNA 6169	TAGTCAAGCGAGACGACACGTAG
	208_R_8_gRNA 6169	AGCTCGTCTTCATGCTCCACTCAA

Supplemental Table S2. Summary of NGS reads count for each tested target site.

Mcsite	CRISPR target	genotype	generation	5- Azacytidine treamtent?	edited reads count
MCsite4	1	WT	T1	No	397
MCsite4	2	WT	T1	No	1106
MCsite4	3	WT	T1	No	1049
MCsite4	4	WT	T1	No	19272
MCsite4	5	WT	T1	No	468
MCsite4	7	WT	T1	No	22338
MCsite4	8	WT	T1	No	253601
MCsite4	9	WT	T1	No	1240
MCsite5	1	WT	T1	No	2495
MCsite5	3	WT	T1	No	21999
MCsite5	4	WT	T1	No	5080
MCsite5	5	WT	T1	No	3772
MCsite5	6	WT	T1	No	6677
MCsite5	7	WT	T1	No	8075
MCsite5	8	WT	T1	No	14552
MCsite4	1	WT	T2	No	440
MCsite4	2	WT	T2	No	1482
MCsite4	3	WT	T2	No	755
MCsite4	4	WT	T2	No	8867
MCsite4	5	WT	T2	No	168
MCsite4	7	WT	T2	No	41693
MCsite4	8	WT	T2	No	88428
MCsite4	9	WT	T2	No	2340
MCsite4	1	WT	T2	Yes	1724
MCsite4	2	WT	T2	Yes	2749
MCsite4	3	WT	T2	Yes	3057
MCsite4	4	WT	T2	Yes	19237
MCsite4	5	WT	T2	Yes	1247
MCsite4	7	WT	T2	Yes	102362
MCsite4	8	WT	T2	Yes	104087
MCsite4	9	WT	T2	Yes	4517
MCsite4	1	CMT3	T1	No	149
MCsite4	2	CMT3	T1	No	152
MCsite4	3	CMT3	T1	No	511
MCsite4	4	CMT3	T1	No	4482
MCsite4	5	CMT3	T1	No	56
MCsite4	7	CMT3	T1	No	15676
MCsite4	8	CMT3	T1	No	94339
MCsite4	9	CMT3	T1	No	240
MCsite5	1	CMT3	T1	No	1795
MCsite5	3	CMT3	T1	No	20003
MCsite5	4	CMT3	T1	No	1991
MCsite5	5	CMT3	T1	No	2117
MCsite5	6	CMT3	T1	No	5290
MCsite5	7	CMT3	T1	No	7377
MCsite5	8	CMT3	T1	No	10643
MCsite4	1	CMT3	T2	No	1277
MCsite4	2	CMT3	T2	No	2138
MCsite4	3	CMT3	T2	No	2709
MCsite4	4	CMT3	T2	No	19925
MCsite4	5	CMT3	T2	No	661
MCsite4	7	CMT3	T2	No	71816
MCsite4	8	CMT3	T2	No	163323
MCsite4	9	CMT3	T2	No	2773
MCsite4	1	CMT3	T2	Yes	2355
MCsite4	2	CMT3	T2	Yes	2555
MCsite4	3	CMT3	T2	Yes	2934
MCsite4	4	CMT3	T2	Yes	10976
MCsite4	5	CMT3	T2	Yes	1539
MCsite4	7	CMT3	T2	Yes	41451
MCsite4	8	CMT3	T2	Yes	129139
MCsite4	9	CMT3	T2	Yes	7043

Read counts used to characterize mutagenesis frequency and mutation outcomes were shown in the “edited reads count” column for each site. The numbers were derived from the sum of all three replicates.

Supplemental Table S3. Oligos for cloning Multicopy CRISPR site (MCsite) and CHLI2 gRNAs.

Oligo Name	Sequence 5' to 3'	Note
92_TRNA_5907	TCGTCTCCGTGCGAGAAAACTGCACCAGCCGGGAATCG	cloning pMOD_B2301 with MCsite4
93_REP_5907	TCGTCTCAGCACTTAAGCTCGTTTTAGAGCTAGAAATAGC	cloning pMOD_B2301 with MCsite4
94_TRNA_518	TCGTCTCCCCATAATGTTGTTGCACCAGCCGGGAATCG	cloning pMOD_B2301 with MCsite3
95_REP_518	TCGTCTCAATGGACAGTCCAGTTTTAGAGCTAGAAATAGC	cloning pMOD_B2301 with MCsite3
96_TRNA_3186	TCGTCTCCCTGCGTTTTGCGTGCACCAGCCGGGAATCG	cloning pMOD_B2301 with MCsite1
97_REP_3186	TCGTCTCAGCAGTCACCATTGTTTTAGAGCTAGAAATAGC	cloning pMOD_B2301 with MCsite1
98_TRNA_3606	TCGTCTCCCTTAAGTGCGAGTGCACCAGCCGGGAATCG	cloning pMOD_B2301 with MCsite2
99_REP_3606	TCGTCTCATAAGCTCTGGTTGTTTTAGAGCTAGAAATAGC	cloning pMOD_B2301 with MCsite2
100_TRNA_715	TCGTCTCCAAGTGTTCGGGTTGCACCAGCCGGGAATCG	cloning pMOD_B2301 with MCsite7
101_REP_715	TCGTCTCAACTTTTCCGGTTGTTTTAGAGCTAGAAATAGC	cloning pMOD_B2301 with MCsite7
102_TRNA_6169	TCGTCTCCATAGTCCACCTATGCACCAGCCGGGAATCG	cloning pMOD_B2301 with MCsite5
103_REP_6169	TCGTCTCACTATGCAGTCAAGTTTTAGAGCTAGAAATAGC	cloning pMOD_B2301 with MCsite5
104_TRNA_6334	TCGTCTCCTTCGTCCATTGATGCACCAGCCGGGAATCG	cloning pMOD_B2301 with MCsite6
105_REP_6334	TCGTCTCACGAACTGCCGCTGTTTTAGAGCTAGAAATAGC	cloning pMOD_B2301 with MCsite6
108_TRNA_CHLI2	TCGTCTCCTTATGAATGTCGTGCACCAGCCGGGAATCG	cloning pMOD_B2301 with CHLI2 and an Mcsite
109_REP_CHLI2	TCGTCTCAATAACAGAGACAGTTTTAGAGCTAGAAATAGC	cloning pMOD_B2301 with CHLI2 and an Mcsite
112_TRNA_term	TGCTCTTCTGACTGCACCAGCCGGGAATCG	universal oligo for cloning pMOD_B2301
113_o35s_prom	TGCTCTTCGCGCATGGAGTCAAAGATTCAA	universal oligo for cloning pMOD_B2301

Supplemental Dataset S1. Characterization of the sequences, DNA methylation, chromatin accessibility, and chromatin states for the 7,971 candidate CRISPR target sites identified.

Supplemental Dataset S2. Annotations of Multicopy CRISPR site (MCsite) 4 and 5. Characterization of the 7 candidate MCsite chromosomal locations, DNA methylation domain, chromatin accessibility, chromatin state, gene annotation, and RNA detection.

Supplemental Dataset S3. Plant Chromatin State Database (PCSD) files for multicopy CRISPR sites (MCsite) 4 and 5. Characterization of the 1 kb region (500 bp upstream and 500 bp downstream) flanking the CRISPR-Cas9 cut site. For each dataset, the values for each individual nucleotide flanking the CRISPR cut site in the 1kb window (500 bp upstream and 500 bp downstream) were quantified by calculating the sum.

Supplemental Dataset S4. PRJNA795172 accession key.

SI References

1. K. Katoh, J. Rozewicki, K. D. Yamada, MAFFT online service: multiple sequence alignment, interactive sequence choice and visualization. *Brief. Bioinform.* **20**, 1160–1166 (2019).

2. Stroud, Hume, Maxim V. C. Greenberg, Suhua Feng, Yana V. Bernatavichute, and Steven E. Jacobsen. 2013. “Comprehensive Analysis of Silencing Mutants Reveals Complex Regulation of the Arabidopsis Methylome.” *Cell* 152 (1): 352–64.

3. Griffin, Patrick T., Chad E. Niederhuth, and Robert J. Schmitz. 2016. “A Comparative Analysis of 5-Azacytidine- and Zebularine-Induced DNA Demethylation.” *G3* 6 (9): 2773–80.
